# Effect of electrode position of low intensity neuromuscular electrical stimulation on the evoked force in the quadriceps femoris muscle

**DOI:** 10.1186/s13104-017-2630-9

**Published:** 2017-07-20

**Authors:** Kohei Watanabe, Shuhei Kawade, Toshio Moritani

**Affiliations:** 1 Laboratory of Neuromuscular Biomechanics, School of International Liberal Studies, Chukyo University, Yagotohonmachi, Showa-ku, Nagoya, 466-8666 Japan; 2 MTG Co., Ltd., Nagoya, Japan; 3 Faculty of Sociology, Kyoto Sangyo University, Kyoto, Japan

**Keywords:** Quadriceps femoris muscles, Knee extensor, Electrode location, Stimulation parameter, Innervation zone, Electrical muscle stimulation

## Abstract

**Objective:**

The present study aimed to test the effect of the electrode position and inter-electrode distance on the evoked force by neuromuscular electrical stimulation (NMES) with a low current intensity and a single pair of electrodes. Knee extensor forces during NMES to quadriceps femoris muscles were compared among four different electrode configurations in seven healthy men. Electrodes were located at 10 cm proximal and 15 cm distal (P10-D15), 10 cm proximal and 10 cm distal (P10-D10), 5 cm proximal and 15 cm distal, and 5 cm proximal and 10 cm distal (P5-D10) to the center of the longitudinal axis of the quadriceps femoris muscles.

**Results:**

The evoked force–time area for P5-D10 was significantly higher than those for P10-D15 and P10-D10 (*p* < 0.05). When using NMES devices with a low current intensity, a shorter inter-electrode distance and relatively distal locations can promote greater evoked forces in the quadriceps femoris muscles.

## Introduction

Neuromuscular electrical stimulation (NMES) has been used to prevent muscle atrophy and promote energy metabolism for clinical purposes [1–5]. It is known that physiological responses during NMES is influenced by electrode configurations, such as distance between stimulation electrodes. For example, Vieira et al. [6] demonstrated that an increase in the electrode distance along the longitudinal axis of the muscles leads to a greater evoked force in the individual muscle components of the quadriceps femoris muscles [6]. They suggested that the greater inter-electrode distances can stimulate a larger number of scattered innervation zones along the muscle.

Recently, portable NMES units are widely available to the general population [4]. However, very few studies have been reported on the optimum conditions for commercially developed NMES devices. For example, their current intensity is limited to a low level, while previous studies used a high current intensity, such as 60–100 mA [6], for experimental and/or clinical applications. We thus suggest that some of the results observed in previous experimental and clinical studies cannot be applied to the physiological response during NMES with commercial devices. Moreover, previous studies [6, 7] placed a bipolar electrode pair for individual muscle components of the quadriceps femoris muscle group. To develop a product for the market, a decrease in the number of electrode pairs is important for user’s convenience and to minimize the price of the product. For effective and/or comfortable applications of NMES for the general public, optimum NMES conditions should be investigated with a limited current intensity and a limited number of electrode pairs.

The present study aimed to test the effect of the electrode position and inter-electrode distance on the evoked force in quadriceps femoris muscles by NMES with a low current intensity and a pair of electrodes.

## Main text

### Methods

Seven healthy young men (age: 22.3 ± 4.4 years, height: 170.9 ± 6.9 cm, body mass: 62.0 ± 7.1 kg) volunteered for the present study. All subjects gave written informed consent for the study after receiving a detailed explanation of the purposes, potential benefits, and risks associated with participation. All subjects were healthy with no history of any musculoskeletal or neurological disorders. All study procedures were conducted in accordance with the Declaration of Helsinki and research code of ethics of Chukyo University, and were approved by the Committee for Human Experimentation of Chukyo University.

**Fig. 1:**
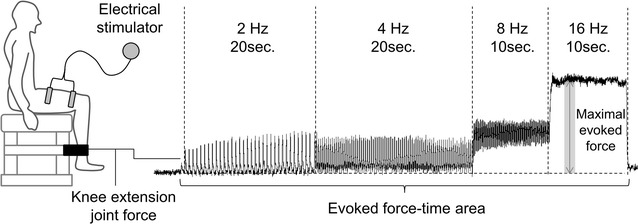
Experimental setting and evoked knee extension joint force during neuromuscular electrical stimulation

**Fig. 2:**
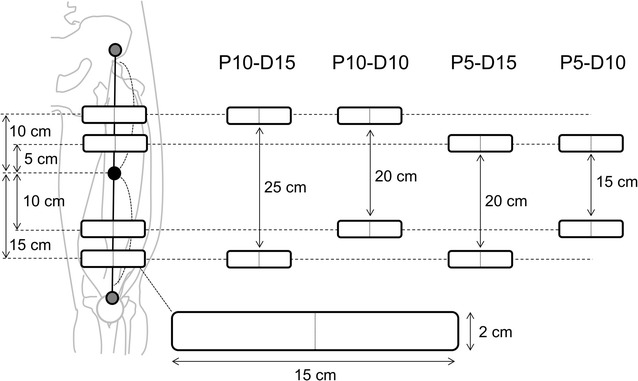
Electrode locations for neuromuscular electrical stimulation

Quadriceps femoris muscles of the right leg were stimulated with two self-adhesive electrodes (2 × 15 cm). We used the custom-made stimulator based on a commercially developed device (Sixpad-Body fit, MTG Ltd. Nagoya, Japan). The stimulation protocol involved 20 s of 2 Hz, 20 s of 4 Hz, 10 s of 8 Hz, and 10 s of 16 Hz (Fig. 1) with the maximum current intensity of the device. We selected lower stimulation frequencies to minimized effect of muscle fatigue. Biphasic square current pulses with a 100 μs duration were applied. The maximal electrical potential and current intensity of this device were 50 V and 4.85 mA, respectively. Two electrodes were placed at various points along the muscle on a reference line (Fig. 2). The reference line was the line between the anterior superior iliac spine and superior edge of the patella. Also, we defined the center of this reference line as the reference point. Four different electrode locations were used: 10 cm proximal and 15 cm distal to the reference point (P10-D15), 10 cm proximal and 10 cm distal from reference point (P10-D10), 5 cm proximal and 15 cm distal from reference point (P5-D15), and 5 cm proximal and 10 cm distal from reference point (P5-D10). Inter-electrode distances were 25 cm for P10-D15, 20 cm for P10-D10, 20 cm for P5-D15, and 15 cm for P5-D10. The center of electrodes was set on the reference line. Self-adhesive electrodes of 2 × 15 cm were used for stimulations. Four configurations with different electrode positions were randomly applied with a 5 min rest interval.

During NMES, the subjects were comfortably seated in a custom-made dynamometer (Takei Scientific Instruments Co. Ltd. Niigata, Japan) with the right leg fixed to a force transducer (LU-100KSE; Kyowa Electronic Instruments, Tokyo, Japan). The force evoked by NMES in the quadriceps femoris muscles was measured as the knee extension force from the force transducer. The knee extension force was sampled at 1000 Hz using A-D converter (Power Lab 16/35; AD Instruments, Melbourne, Australia) and filtered with a low pass filter at a 20 Hz cut-off frequency (4th order Butterworth filter). The present study analyzed two parameters for the evoked force during NMES: (1) maximal evoked force during NMES at 16 Hz, and (2) summation of the evoked force–time area during NMES at 2, 4, 8, and 16 Hz (Fig. 1). The maximal evoked force was an averaged value of the evoked force over 1 s from the time just after the evoked force reaches a plateau during the sustained phase of NMES at 16 Hz. For each subject, these two parameters at each electrode position were normalized by peak values under all configurations.

Before NMES, the maximal voluntary contraction (MVC) during isometric knee extension was measured using our previously reported procedure [8]. Briefly, the subjects were asked to gradually increase their knee extension force from the baseline to maximum in 2–3 s and then sustain it maximally for 2 s. The MVC force was used to quantify the evoked force for each subject.

We used mom-parametric tests since the sample size was not large and data distribution was partly non-Gaussian. The effect of the electrode position on the maximal evoked force and evoked force–time area was assessed by the Friedmann test. Also, the parameters for P5-D10 were compared with the three other configurations by the Wilcoxon test since the highest values were observed for P5-D10 among these parameters. The level of significance was set at 0.05/number of compared pairs, i.e., 0.05/3 = 0.016 in the case of comparison with three configurations in the Wilcoxon test. Statistical analyses were performed using SPSS software (version 15.0; SPSS, Tokyo, Japan).

## Results

The mean evoked knee extension forces during NMES at 16 Hz were 6.5 ± 5.5, 11.6 ± 12.8, 10.7 ± 8.1, and 13.6 ± 12.1% of MVC for P10-D15, P10-D10, P5-D15, and P5-D10, respectively. Also, maximal evoked knee extension forces during NMES at 16 Hz were 7.1 ± 5.6, 12.2 ± 13.0, 11.8 ± 9.7, and 15.0 ± 12.1% of MVC for P10-D15, P10-D10, P5-D15, and P5-D10, respectively.

**Fig. 3:**
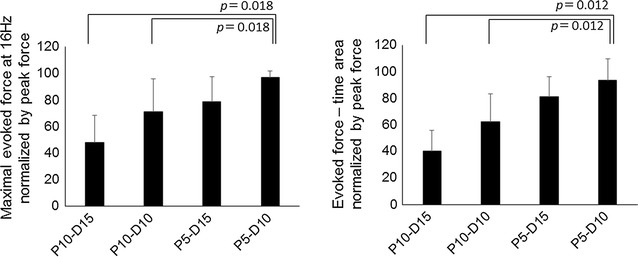
Maximal evoked force at 16 Hz and evoked force–time area during neuromuscular electrical stimulation for each configuration. These values were normalized by the peak value across four configurations for each subject

The maximal evoked force and evoked force–time area were significantly changed with the electrode position (*p* = 0.001 for the maximal evoked force and *p* = 0.0001 for the evoked force–time area, Friedmann test) (Fig. 3). The evoked force–time area for P5-D10 was significantly larger than those for P10-D15 and P10-D10 (*p* = 0.012 for each) (Fig. 3).

## Discussion

The present study demonstrated that the evoked force during NMES is influenced by the electrode position, and a closer inter-electrode distance induces a greater force. This result is not consistent with that of a previous study [6], but this can be explained by a difference in the current intensity. While Vieira et al. [6] applied a 60–100 mA current intensity, our device generated a 4.85 mA current intensity. Since innervation zones for the quadriceps femoris muscles are regionally distributed along the muscle [9–11], the greater inter-electrode distance would stimulate a larger number of innervation zones when the current intensity is high enough to stimulate the innervation zone and/or muscle under and between electrodes. Theoretically, an increase in the inter-electrode distance would decrease the current density within the underlying tissue. Therefore, the greatest force was applied with a shorter inter-electrode distance for NMES with a low current intensity in the present study.

Although the inter-electrode distance was the same, a difference in the evoked force was shown between P10-D10 and P5-D15 (Fig. 3). This suggests that the electrode position is also important in addition to the inter-electrode distance during NMES. P5-D15 is an electrode position 5 cm distal to P10-D10. Since the innervation zones of the vastus lateralis [10, 11] and vastus medialis [9–11] muscles are also distributed near the patella and the muscle belly of the vastus medialis muscle is located in a relatively distal part of the thigh [12, 13], P5-D15 may be able to stimulate more muscle fibers in these two muscles.

In conclusion, our study suggests that when the NMES with low current intensity is applied for the quadriceps femoris muscles, shorter inter-electrode distance (15–20 cm) and 5 cm proximal and 10 or 15 cm distal from the center of longitudinal axis of the muscle group of electrode locations could induce greater evoked force. From the muscle length for our subjects, these recommended electrode location is corresponded with 38.6–72.9/84.3% of length of whole quadriceps femoris muscle group. In commercial NMES devices with low current intensity which are used in general populations, optimum conditions could be different from the NMES devices with high current intensity for scientific or clinical purposes [14]. Our findings might be applied in design of commercial NMES devices with low current intensity or to people that available current intensity is limited to low level such as patients or elderly.

### Limitations

Our experiment was performed in small sample size. Also, we selected a fixed stimulation parameter and the four electrode configurations from various combinations. Since we aimed to apply our results to commercially developed NMES devices, current intensity, pulse duration, and frequency were matched for the parameters used in commercially developed materials. Large number of trials in NMES would induce muscle fatigue. Therefore, we used a limited number of electrode configurations which were selected based on preliminary test and innervation pattern of quadriceps femoris muscles [9–11].

## Abbreviations

MVC: maximal voluntary contraction
NMES: neuromuscular electrical stimulation
